# Risk factors related to the severity of COVID-19 in Wuhan

**DOI:** 10.7150/ijms.47193

**Published:** 2021-01-01

**Authors:** Chen Zhao, Yan Bai, Cencen Wang, Yanyan Zhong, Na Lu, Li Tian, Fucheng Cai, Runming Jin

**Affiliations:** 1 Department of Pediatric, Union Hospital, Tongji Medical College, Huazhong University of Science and Technology, 1277 Jiefang Road, Wuhan, 430022, P.R. China.; 2 Huazhong University of Science and Technology Hostipal. Luoyu Road 1037, Wuhan, 430074, P.R China.

**Keywords:** COVID-19, moderate cases, severe prognosis, risk factors, severity, prognostic prediction, machine learning

## Abstract

**Objective:** To evaluate the characteristics at admission of patients with moderate COVID-19 in Wuhan and to explore risk factors associated with the severe prognosis of the disease for prognostic prediction.

**Methods:** In this retrospective study, moderate and severe disease was defined according to the report of the WHO-China Joint Mission on COVID-19. Clinical characteristics and laboratory findings of 172 patients with laboratory-confirmed moderate COVID-19 were collected when they were admitted to the Cancer Center of Wuhan Union Hospital between February 13, 2020 and February 25, 2020. This cohort was followed to March 14, 2020. The outcomes, being discharged as mild cases or developing into severe cases, were categorized into two groups. The data were compared and analyzed with univariate logistic regression to identify the features that differed significantly between the two groups. Based on machine learning algorithms, a further feature selection procedure was performed to identify the features that can contribute the most to the prediction of disease severity.

**Results:** Of the 172 patients, 112 were discharged as mild cases, and 60 developed into severe cases. Four clinical characteristics and 18 laboratory findings showed significant differences between the two groups in the statistical test (*P*<0.01) and univariate logistic regression analysis (*P*<0.01). In the further feature selection procedure, six features were chosen to obtain the best performance in discriminating the two groups with a linear kernel support vector machine. The mean accuracy was 91.38%, with a sensitivity of 0.90 and a specificity of 0.94. The six features included interleukin-6, high-sensitivity cardiac troponin I, procalcitonin, high-sensitivity C-reactive protein, chest distress and calcium level.

**Conclusions:** With the data collected at admission, the combination of one clinical characteristic and five laboratory findings contributed the most to the discrimination between the two groups with a linear kernel support vector machine classifier. These factors may be risk factors that can be used to perform a prognostic prediction regarding the severity of the disease for patients with moderate COVID-19 in the early stage of the disease.

## Introduction

COVID-19 was initially reported in Wuhan, China, in December 2019 and rapidly spread to all other provinces in China and throughout the world 1, 2. Without specific treatment or prevention options for COVID-19, such as targeted antiviral drugs and vaccines, China has focused on isolation, quarantine, social distancing, and community containment to contain the outbreak 3. By May 18, 2020, there were 84,494 confirmed cases of COVID-19 and 4,645 deaths in China and 4,534,327 confirmed cases and 307,202 deaths outside of China 4. The pandemic of COVID-19 has raised wide public concern and imposed a heavy burden on global health care systems because approximately 15-20% of patients develop severe interstitial pneumonia 5. In addition, COVID-19 patients admitted to ICUs experienced higher mortality (38%) than non-ICU patients (4%) 6. A mortality rate of 50-60% was reported in patients developing ARDS and requiring invasive mechanical ventilation therapy in the ICU 7.

Patients with COVID-19 were divided into mild, moderate, severe, and critical cases 8. Because of the high mortality rate in severe or critical patients 6, 7, early identification of patients' risk of developing into severe or critical cases is important so that patients with a poor prognosis can receive timely intervention and minimize the progression of the disease 9. Therefore, prognostic tools and biomarkers are urgently needed 10, 11. However, most studies have focused mainly on identifying the factors related to death and recovery 12-14. Although some prognostic information has been revealed by using univariate- or multivariate analyses based on prior clinical knowledge or evidence 9, 15-21, these studies have not paid enough attention to feature selection in multivariate prognostic prediction modeling. As a result, the published prognostic prediction tools may not make the most of patient data to perform prognostic prediction modeling.

This study built a multivariate prognostic prediction model to predict the risk of developing severe cases among patients with moderate COVID-19. With the patients' characteristics at admission and outcomes, a feature selection procedure based on machine learning algorithms was conducted to identify the features contributing the most to distinguishing between the two groups. These features were then chosen as the risk factors on which to build the prognostic prediction model. We believe that this multivariate prognostic prediction tool will be of considerable value for patients with moderate COVID-19 in isolation or self-quarantine so that they can receive timely intervention and active intensive care to minimize progression of the disease and so that health care agencies can prioritize their services, especially in resource-constrained areas.

## Materials and methods

The ethics committees of Union Hospital, Tongji Medical College, Huazhong University of Science and Technology approved this retrospective study. The requirement for informed consent was waived. This study was conducted in accordance with the Declaration of Helsinki.

### Patients

**Figure 1:**
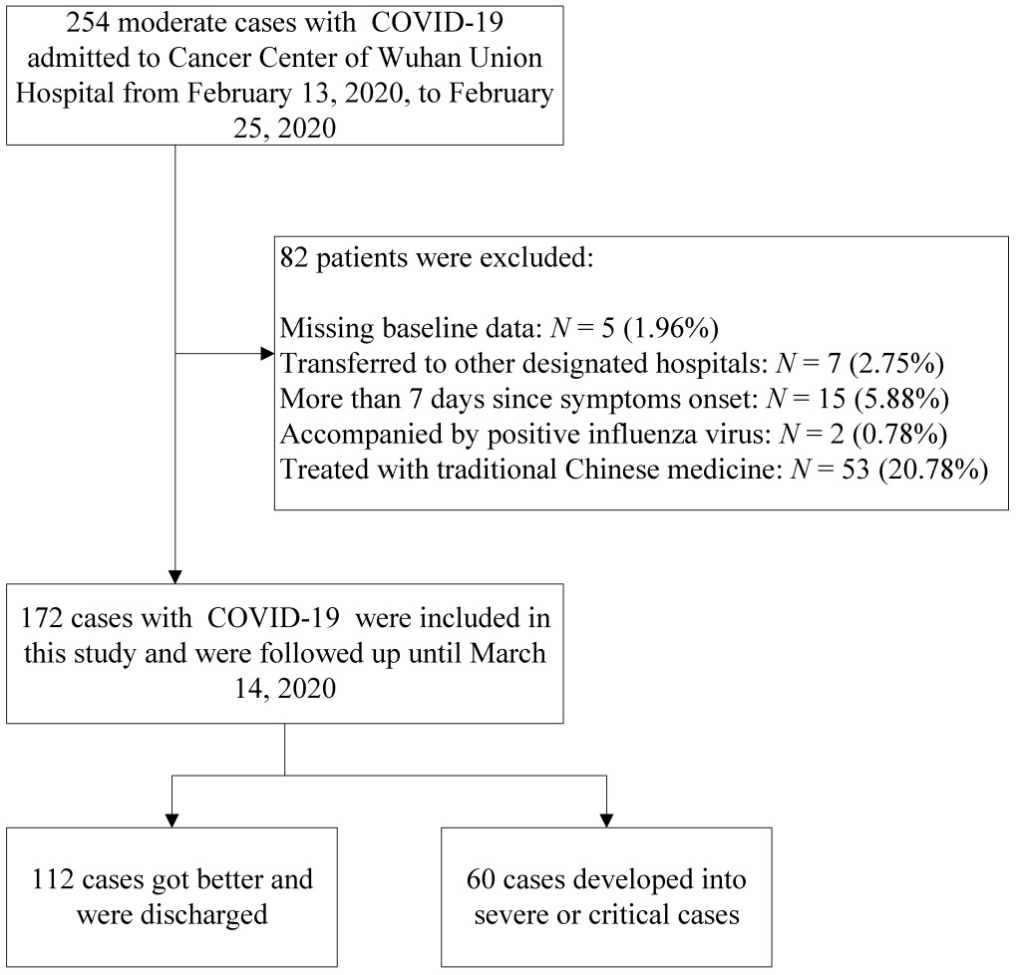
Study population.

In this retrospective, single-center study, a search for patient data in the electronic record system was performed for patients admitted to the Cancer Center of Wuhan Union Hospital between February 13, 2020 and February 25, 2020. According to the report of the WHO-China Joint Mission on COVID-19, patients with COVID-19 were divided into mild (laboratory confirmed, without pneumonia), moderate (laboratory confirmed and with pneumonia), severe (dyspnea, respiratory frequency ≥30 beats per minute (bpm), oxygen saturation (SpO_2_) ≤93%, PaO_2_/FiO_2_ ratio <300, and/or lung infiltrates >50% of the lung field within 24-48 hours), and critical (respiratory failure requiring mechanical ventilation, shock, or other organ failure that requires intensive care) cases 8. Our institution was a designated hospital capable of receiving patients with moderate, severe and critical cases of COVID-19. The inclusion criteria in this study were as follows: (1) patients with laboratory-confirmed COVID-19 according to viral nucleic acid detection using RT-PCR with samples from pharynx swabs; (2) patients who underwent complete laboratory tests (routine blood tests, biochemistry analysis, cytokine tests, immunology tests, and L subset tests) and clinical recording at admission; and (3) patients diagnosed with moderate COVID-19 at admission. The flow diagram of the exclusion criteria is shown in **Figure 1.**

### Treatments

The number of patients with COVID-19 is rapidly growing worldwide, and specialized treatment has not been available in the early stage of the global outbreak. Patients in this study were moderate cases, and their treatments followed the therapeutic principles based on the 2019-nCoV guidelines (Trial Version 5) proposed by the China National Health Commission 22. The basic treatment included antiviral treatment (abidor 200 mg three times daily, orally), antibacterial treatment (moxifloxacin 400 mg once daily, orally), recombinant human interferon α2b (aerosol inhalation) and symptomatic treatment. Some of the moderate cases were treated with traditional Chinese medicine. However, they were not included in this study, as shown in **Figure 1.**

### Data collection

This cohort was followed to March 14, 2020. Patient data were obtained at admission, including demographics, comorbidities, signs and symptoms, and laboratory findings. The assessed comorbidities included hypertension, cardiovascular disease, diabetes, malignancy, cerebrovascular disease, COPD, chronic kidney disease, chronic liver disease, HIV infection, rheumatic disease and hyperuricemia. The laboratory findings were obtained through the complete laboratory tests mentioned earlier. Finally, 172 patients with moderate COVID-19 at admission were included in this study. In addition, 112 cases were discharged as mild cases, whereas 60 cases developed into severe or critical cases.

### Statistical analysis and prognostic prediction modeling

Descriptive statistics were used to describe the demographics, comorbidities, signs and symptoms, and laboratory findings of the 172 moderate cases. Between the two groups, categorical data were compared by using the chi-squared test (Fisher's exact test if the expected count was fewer than 5 for at least one cell). Continuous variables were compared using the independent variable *t*-test (Mann-Whitney *U* test if the data were not normally distributed). Univariate logistic regression models were also built to identify the potential risk factors related to the severe prognosis of COVID-19. As a result, the features that differed significantly between the two groups in the above statistics test (*P*<0.01) and were significant in univariate logistic regression (*P*<0.01) were chosen as candidates for further processing as follows.

First, multivariate logistic regression analysis with L1 regularization was performed with feature standardization. The regression aimed to identify a subset of features from the aforementioned candidate features that could contribute the most to the discrimination between the two groups 23. In addition, a parameter sweep on the *C* parameter was performed in the mentioned L1 regularization. Second, an SVM classifier with a linear kernel was adopted to measure the prediction performance of the top *k* features from the aforementioned subset. The top *k* features were selected according to their coefficients in the previous multivariate logistic regression (*k* ranged from 1 to the size of the subset). Finally, the top *k* features with the highest 5-fold SVM classification accuracy were chosen as the most predictive features to perform the clinical prognostic prediction modeling with a linear kernel SVM model. The predicted probability (*p*_i_) of a moderate case developing into a severe one was calculated with the following equation: ln [*p*_i_/(1-*p*_i_)] = *a*_0_ + *a*_1_ * *x*_i1_ +…+ *a*_k_ * *x*_ik_. For the *i*th individual, *x*_ik_ was the kth indicator variable in the prognostic prediction model, and *a*_ik_ was the weight for the kth feature. *a*_0_ is the intercept.

The statistical analysis was performed using SPSS 22 (SPSS, Inc., Chicago, IL, USA). SVM modeling and multivariate logistic regression modeling with L1 regularization used Python (Version 3.7) and the scikit-learn package (Version 0.22.1) 24.

## Results

### Clinical characteristics

The basic information about the two groups is summarized in **Table 1.** The median age of the patients was 65 years (IQR 57-71 years). Patients in the severe group were significantly older than those in the mild group (*P*<0.001), with an average age of 70.6 (SD 11.6) and a median age of 64 (IRQ 50-67), respectively. Among the 172 cases, 52.3% of the patients were female. There was a higher female proportion in the mild group than in the severe group (*P*=0.01). Comorbidities were present in 55.2% of the patients, but the difference between the two groups was not significant. Hypertension and diabetes were the most common comorbidities. For each kind of comorbidity, there was no significant difference between the two groups.

As shown in **Table 2**, 15 signs or symptoms were recorded in these moderate cases at admission. Fever, dry cough, and fatigue were the most common initial symptoms (63.4%, 55.2%, and 58.1%, respectively). But fever and dry cough showed no significant difference between the two groups, while fatigue was significantly different. Chest distress and anorexia were significantly more common in the severe group than in the mild group (*P*<0.001). In the univariate logistic regression analysis, age, chest distress, fatigue, and anorexia showed significant differences regarding to the outcomes (*P*<0.01), as shown in **Table 4.**

### Laboratory findings

Laboratory findings on hospital admission are summarized in **Table 3.** Among the 172 patients, 19 laboratory findings showed significant differences between the two groups. Patients in the severe group demonstrated significantly increased WBC, N count, N percentage, AST, LDH, TNI, CK, CK-MB, CysC, ESR, CRP, PCT, IL-6, and IL-10 but significantly decreased L count, L percentage, A/G, ALB, and Ca (*P*<0.01). In the univariate logistic regression analysis, these laboratory findings also showed significant differences regarding to the outcomes (*P*<0.01), except CK-MB, as shown in **Table 4.**

### Prediction model for severe prognosis

**Figure 2:**
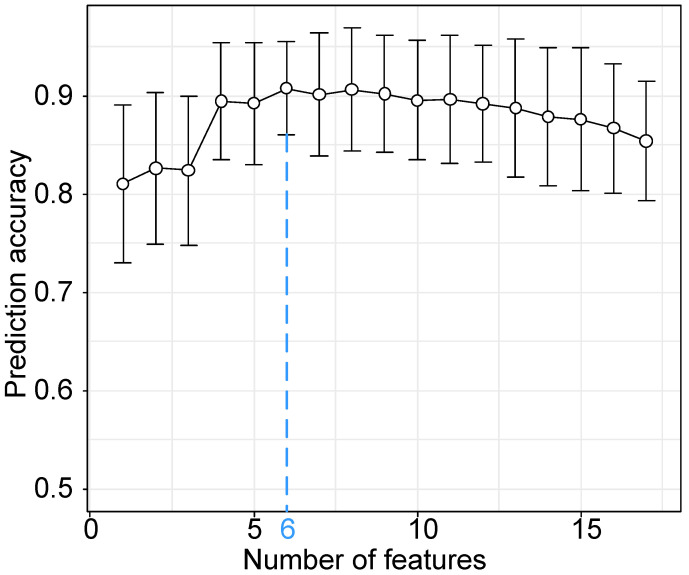
Classification accuracy of the prognostic prediction model with a linear kernel SVM model and the top *k* features. The classification accuracy was measured with 5-fold cross validation. The blue dotted vertical line was drawn at the optimal number 6 with the parameter *C*=3.999 when the classification accuracy reached the highest point 91.38%. The six most predictive features used in the prognostic prediction model were IL-6, TNI, PCT, CRP, chest distress and Ca.

**Figure 3:**
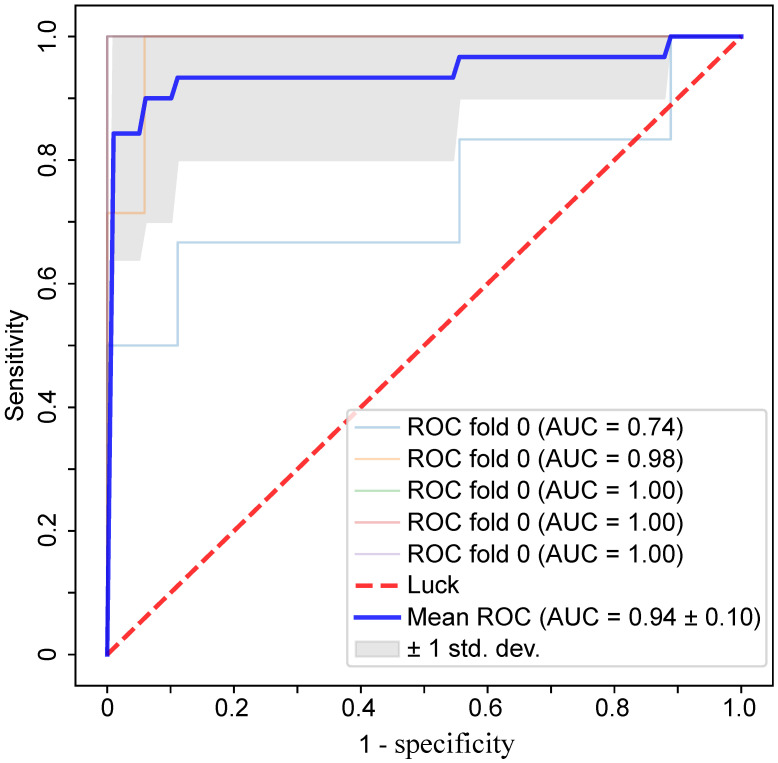
The ROC curves for the prognostic prediction model with a linear kernel SVM and 5-fold cross validation. With the six most predictive features, the mean area under the ROC curve was 0.94, with a sensitivity of 0.90 and a specificity of 0.94.

Based on the results of statistical analysis and univariate logistic regression, four clinical characteristics and 18 laboratory findings differed significantly between the two groups (*P*<0.01) and were significant in univariate logistic regression (*P*<0.01), as shown in **Table 4.** In the further feature selection procedure, these features were used to perform multivariate logistic regression with L1 regularization and feature standardization. With a parameter *C*=3.999 in the regularization, 17 features were finally selected as shown in **Table 4.** Eventually, the top six features ranked by regression coefficients were chosen as the most predictive features with which to build a prognostic prediction model for severity for patients with moderate COVID-19. The highest prediction accuracy of 91.38% was reached with the selected six features and a linear kernel SVM with 5-fold cross validation, as shown in **Figure 2.** The SVM model for prognostic prediction had a sensitivity of 0.90 and a specificity of 0.94, with a mean area under the ROC curve of 0.94, as shown in **Figure 3.** In the formula for the multivariate prognostic prediction model, the intercept *a*_0_ was -0.14, and the feature weights for the six most predictive features are shown in **Table 4.**

## Discussion

The number of patients infected with COVID-19 is still increasing rapidly worldwide. However, specialized and effective treatment is not yet available. Therefore, the early identification of a moderate case's risk developing into a severe or critical one is of great importance due to the high mortality rate among severe and critical cases 6, 7. Timely intervention and active intensive care can help to minimize the progression of the disease. Therefore, paying attention to prognostic prediction for moderate cases can be important. We enrolled 172 patients hospitalized with laboratory-confirmed COVID-19 and diagnosed with moderate COVID-19 at admission. The patient data were systematically analyzed. As a result, six risk factors were identified as the most predictive ones with which to perform prognostic prediction of severity for patients with moderate COVID-19.

Consistent with previous research results, patients in the severe group were significantly older than those in the mild group. Older patients presented more comorbidities and were more likely to develop severe or critical COVID-19 5, 6, 25, 26. The proportion of severe cases in males was 61.7%, which was significantly higher than that in females (38.3%), as reported in a recent study 9. Several studies concerning comorbid disease with COVID-19 suggested that adequate attention should be paid to comorbidity 18, 26, 27. In our study, the most common comorbidity was hypertension (36.6%, 63/172), followed by diabetes (15.7%, 27/172) and cardiovascular disease (9.9%, 17/172). Due to the limited sample size, there were no significant differences in comorbidities between the two groups. For all the moderate cases in this study, chest distress, fatigue, and anorexia were significantly different between the two groups, which was consistent with previous reports 28, 29.

In addition, in laboratory findings of the moderate cases at admission, significantly higher levels of WBC, N count, N percentage, AST, LDH, TNI, CK, CK-MB, CysC, ESR, CRP, PCT, IL-6, and IL-10 were found in the severe group compared with the mild group. L count, L percentage, A/G, ALB, and Ca, however, were found to be at lower levels in the severe group. The severity of COVID-19 infection may activate neutrophils to produce an immune response to the virus and cause a cytokine storm. Furthermore, considering that aging is related to decreased immune competence 30, elderly patients who died consequently may be due to a weak immune response 16. There were no significant differences in the L subsets between the two groups, possibly because of the limited sample size. However, some impressive different results were reported in previous research, indicating that CD3^+^ and CD4^+^ T cells might protect patients from developing ARDS 16.

In this study, a feature selection procedure based on machine learning algorithms was performed to identify the most predictive features for a multivariate prognostic prediction of severity. Feature selection was an essential step for multivariate modeling. In previous studies, this was mainly implemented by performing univariate statistical analysis or with prior clinical knowledge or evidence 9, 15, 17, 19. In our study, a two-step feature selection procedure was performed based completely on patient data. Finally, one clinical characteristic (chest distress) and five laboratory findings (IL-6, TNI, PCT, CRP, Ca) were identified as the most predictive risk factors for prognostic prediction of severity for patients with moderate COVID-19. Without adequate knowledge about COVID-19, information completely extracted from the data may be of great value to perform valid prognostic prediction.

The level of IL-6 was mildly elevated or within the normal range in the mild group but markedly elevated in the severe group. Elevated cytokines were likely produced by highly inflammatory macrophages that were implicated in a cytokine storm 31. Myocardial damage with biomarker elevations was a prominent feature in COVID-19 and was related to a worse prognosis 32, 33. TNI, as one of the most predictive factors in our prognostic prediction model, was used to evaluate patients with suspected acute coronary syndrome 34. TNI level in plasma and CRP level were positively and significantly related 35. This implies that the pathological process of myocardial damage and inflammation might have a close relationship in the course of COVID-19 disease. Chest distress was an important factor that could contribute to the prognostic prediction of severity in this study and was reported as one of the most common symptoms in COVID-19 28. Increasing PCT level was reported in the discrimination between mild and moderate cases 36. In our study, PCT was identified as a risk factor for prognostic prediction of severity. Lower level of Ca was recognized as a predictor of severity in our prognostic prediction model. This was an interesting result that has not yet been reported.

There are also some limitations in our research. First, a larger sample size may result in a more convincing prognostic prediction model with the feature selection procedure based on machine learning algorithms. Due to the limited size, the comorbidities and some signs and symptoms showed no significant difference between the two groups. Moreover, age was not one of the most predictive factors related to severity in this cohort of moderate cases, but age was an important risk factor in prognosis in previous research 9. Second, the temporal changes in patient characteristics from admission to outcome were not included in this study because most of the tests were performed only once at admission. The prognosis may benefit from information on temporal changes 19. Third, the patients' stage of disease progression at admission, such as time since symptom onset and exposure history, was not considered as a candidate predictor. This information relied on patients' memory and might have been affected by recall bias. Fourth, the data in this study were from an outbreak, so these might differ in a nonoutbreak situation. However, since we included as many COVID-19 cases as possible in our hospital, we believe our study population is representative of cases diagnosed and treated in Wuhan.

## Conclusions

With the data collected at admission, one clinical characteristic (chest distress) and five laboratory findings (IL-6, TNI, PCT, CRP, Ca) showed the best performance in discriminating between the two groups with a linear kernel SVM model. They may be risk factors that can be used to perform a prognostic prediction of severity for patients with moderate COVID-19 in the early stage of the disease and thus help minimize the progression of the disease.

## Figures and Tables

**Table 1:** Comparison of epidemiology and baseline characteristics between mild and severe outcomes of patients with COVID-19

Characteristics	Total cases (n=172)	Mild (n=112)	Severe (n=60)	*P* value
**Demographics**				
Age (years)	65 (57-71)	64 (50-67)	70.6 (11.6)	<**0.001*^a^***
**Gender**				
Female	90 (52.3%)	67 (59.8%)	23 (38.3%)	0.01*^b^*
Male	82 (47.7%)	45 (40.2%)	37 (61.7%)	
**Comorbidities**				
Background Diseases	95 (55.2%)	57 (50.9%)	38 (63.3%)	0.107*^b^*
Hypertension	63 (36.6%)	38 (33.9%)	25 (41.7%)	0.318*^b^*
Cardiovascular disease	17 (9.9%)	7 (6.3%)	10 (16.7%)	0.033*^b^*
Diabetes	27 (15.7%)	12 (10.7%)	15 (25.0%)	0.016*^b^*
Malignancy	7 (4.1%)	4 (3.6%)	3 (5.0%)	0.694
Cerebrovascular disease	5 (2.9%)	1 (0.9%)	4 (6.7%)	0.049
COPD	4 (2.3%)	2 (1.8%)	2 (3.3%)	0.609
Chronic kidney disease	3 (1.7%)	1 (0.9%)	2 (3.3%)	1.000
Chronic liver disease	3 (1.7%)	2 (1.8%)	1 (1.7%)	1.000
HIV infection	1 (0.6%)	1 (0.9%)	0 (0)	1.000
rheumatic disease	3 (1.7%)	3 (2.7%)	0 (0)	0.552
hyperuricemia	4 (2.3%)	1 (0.9%)	3 (5.0%)	0.119

**Note**: Categorical data are n (%). Continuous data are the mean (SD) if following a normal distribution according to the Shapiro-Wilk test (*P*<0.01); otherwise, the median (lower quartile-upper quartile) is shown. *a:* Mann-Whitney *U* test; *b:* chi-squared test; otherwise, Fisher's exact test, owing to an expected count less than five for at least one cell.

**Table 2:** Comparison of signs and symptoms between mild and severe outcomes of patients with COVID-19

Signs and symptoms	Total cases (n=172)	Mild (n=112)	Severe (n=60)	*P* value
Fever	109 (63.4%)	71 (63.4%)	38 (63.3%)	1*^a^*
Dry cough	95 (55.2%)	64 (57.1%)	31 (51.7%)	0.523*^a^*
Expectoration	30 (17.4%)	19 (17.0%)	11 (18.3%)	0.836*^a^*
Pharyngalgia	9 (5.2%)	6 (5.3%)	3 (5.0%)	1
Runny nose	7 (4.1%)	4 (3.6%)	3 (5.0%)	0.696
Headache	7 (4.1%)	5 (4.5%)	2 (3.3%)	1
Chest distress	59 (34.3%)	24 (21.4%)	35 (58.3%)	**<0.001**
Pectoralgia	4 (2.3%)	3 (2.7%)	1 (1.7%)	1
Myalgia	38 (22.1%)	24 (21.4%)	14 (23.3%)	0.848*^a^*
Fatigue	100 (58.1%)	55 (49.1%)	45 (75%)	**0.001*^a^***
Anorexia	75 (43.6%)	37 (33.0%)	38 (63.3%)	**<0.001*^a^***
Diarrhea	15 (8.7%)	9 (8.0%)	6 (10%)	0.778*^a^*
Emesis	7 (4.1%)	5 (4.5%)	2 (3.3%)	1
Ageusia	21 (12.2%)	13 (11.6%)	8 (13.3%)	0.808*^a^*
Anosmia	32 (18.6%)	19 (17.0%)	13 (21.7%)	0.538*^a^*

**Note**: Categorical data are n (%); *a:* Chi-squared test; otherwise, Fisher's exact test, owing to expected count less than five for at least one cell.

**Table 3:** Comparison of laboratory findings between mild and severe outcomes of patients with COVID-19

Laboratory findings	Total cases (n=172)	Mild (n=112)	Severe (n=60)	*P* value
**Hematologic parameters**				
WBC (G/L)	5.8 (4.5-7.3)	5.4 (4.4-6.9)	6.5 (5.1-7.9)	**0.006**
N percentage (%)	64.9 (56.2-73)	60.6 (54.6-66.6)	74.8 (65.5-86.6)	**<0.001**
N (G/L)	3.56 (2.75-5.02)	3.32 (2.52-4.31)	4.69 (3.38-6.51)	**<0.001**
L percentage (%)	22.8 (10.8)	26.8 (8.9)	15.4 (10.1)	**<0.001*^a^***
L (G/L)	1.29 (0.86-1.67)	1.51 (1.11-1.77)	0.81 (0.53-1.29)	**<0.001**
Hb (g/L)	120 (15)	121 (12)	119 (19)	0.535*^a^*
PLT (G/L)	216 (165-280)	224 (180-291)	208 (92)	0.036
**Biochemical parameters**				
TB (µmol/L)	11.5 (9.1-14.1)	11.2 (3.5)	12.2 (9.8-18)	0.01
ALT (U/L)	28 (18-49)	27 (18-46)	34 (18-52)	0.429
AST (U/L)	26 (20-37)	23 (20-30)	32 (24-47)	**0.001**
A/G	1.20 (1.10-1.55)	1.40 (1.20-1.70)	1.10 (0.90-1.28)	**<0.001**
ALB (g/L)	36.0 (32.1-40.3)	38.2 (4.6)	31.1 (28.0-35.0)	**<0.001**
GLB (g/L)	27.8 (24.6-32.2)	28.0 (5.1)	30.3 (6.2)	0.01*^a^*
ALP (U/L)	77 (63-99)	76 (62-98)	84 (69-106)	0.096
LDH (U/L)	200 (167-273)	181 (164-230)	273 (211-407)	**<0.001**
TNI (ng/L)	2.80 (1.40-8.18)	1.80 (1.00-3.75)	10.1 (3.1-170)	**<0.001**
CK (U/L)	56.5 (39.8-87.3)	51 (39-73)	75 (41-142)	**0.003**
CK-MB (ng/mL)	0.50 (0.30-1.00)	0.40 (0.30-0.68)	0.95 (0.50-2.80)	**<0.001**
CysC (mg/L)	1.13 (0.95-1.39)	1.08 (0.91-1.23)	1.25 (1.02-1.78)	**0.001**
BUN (mmol/L)	4.30 (3.50-5.38)	4.20 (3.49-5.00)	4.50 (3.66-9.61)	0.012
CREA (µmol/L)	66 (57-81)	65 (56-75)	75 (58-92)	0.027
TG (mmol/L)	1.38 (1.01-1.86)	1.41 (1.02-1.91)	1.32 (1.00-1.77)	0.389
Na (mmol/L)	139 (137-141)	140 (138-141)	139 (6)	0.161
K (mmol/L)	4.14 (3.72-4.50)	4.15 (0.45)	4.12 (0.86)	0.753*^a^*
Ca (mmol/L)	2.38 (0.17)	2.30 (0.15)	2.13 (0.16)	**<0.001*^a^***
ESR (mm/h)	38 (20-59)	37.4 (22.3)	51.3 (27.1)	**0.004*^a^***
CRP (mg/L)	4.2 (2.9-36.8)	3.1 (1.0-8.1)	76.4 (14.0-132.8)	**<0.001**
PCT (µg/L)	0.13 (0.09-0.13)	0.13 (0.06-0.13)	0.13 (0.13-0.21)	**<0.001**
**Cytokine levels**				
IL-6 (pg/ml)	11.1 (5.9-33.1)	8.1 (5.4-11.5)	54.7 (27.5-88.4)	**<0.001**
TNF-α (pg/ml)	3.55 (2.73-5.47)	3.72 (2.79-5.80)	3.28 (2.51-4.90)	0.181
IL-4 (pg/ml)	2.83 (2.08-3.76)	3.14 (2.11-3.85)	2.53 (2.06-3.34)	0.063
IL-2 (pg/ml)	2.97 (2.56-3.97)	3.12 (2.60-4.06)	2.87 (2.56-3.66)	0.242
IL-10 (pg/ml)	4.27 (3.43-5.22)	4.10 (3.09)	4.71 (3.66-6.10)	**0.006**
IFN-γ (pg/ml)	2.87 (2.13-3.50)	2.97 (2.09-3.51)	2.63 (2.15-3.50)	0.635
**Lymphocyte subpopulation**				
CD4^+^/CD8^+^	1.93 (1.49-2.84)	1.86 (1.47-2.68)	2.16 (1.53-3.30)	0.101
CD3^+^ (%)	75.5 (68.2-80.8)	73.9 (9.4)	75.2 (66.2-81.6)	0.826
CD4^+^ (%)	45.4 (10.7)	45.2 (9.3)	45.6 (13.1)	0.824*^a^*
CD8^+^ (%)	23.7 (17.8-29.3)	24.7 (8.3)	19.8 (15.0-30.3)	0.078
B lymphocyte (%)	11.1 (8.2-15.5)	12.1 (5.2)	10.1 (8.3-16.2)	0.650
NK (%)	7.6 (4.7-12.0)	7.2 (4.9-12.0)	9.5 (3.7-12.0)	0.491
**Immuneglobulin**				
IgG (g/L)	10.9 (9.3-12.8)	11.5 (9.8-13.2)	10.2 (8.1-11.6)	0.052
IgA (g/L)	2.43 (1.70-3.04)	2.56 (1.15)	2.56 (1.35)	0.991*^a^*
IgM (g/L)	1.01 (0.75-1.33)	1.05 (0.80-1.36)	0.86 (0.56-1.10)	0.092
IgE (IU/ml)	33.8 (13.4-102.5)	30.3 (11.3-64.0)	47.9 (16.8-182.7)	0.039
**Complement**				
C3 (g/L)	0.87 (0.80-0.98)	0.86 (0.13)	0.92 (0.21)	0.203*^a^*
C4 (g/L)	0.21 (0.07)	0.20 (0.06)	0.24 (0.10)	0.027

**Note**: Continuous data are the mean (SD) if following a normal distribution according to the Shapiro-Wilk test (*P*<0.01); otherwise, median (lower quartile-upper quartile); *a:* Independent variable *t-*test; otherwise, Mann-Whitney *U* test.

**Table 4:** Risk factors associated with the severity of patients with COVID-19

Demographics and clinical characteristics	Univariable OR (95% CI)	*P* value	Regression coefficient	Feature weight
Age, years*	1.086 (1.050-1.124)	<0.001	0.08	
Chest distress	5.133 (2.592-10.168)	<0.001	**1.05*^a^***	**0.41**
Fatigue	3.109 (1.557-6.210)	0.001	0.55	
Anorexia	3.501 (1.816-6.749)	<0.001	0.20	
**Laboratory findings**				
WBC (G/L)*	1.27 (1.08-1.49)	0.003		
N percentage (%)*	1.10 (1.07-1.14)	<0.001	0.19	
N (G/L)*	1.27 (1.09-1.48)	0.002	0.29	
L percentage (%)*	0.88 (0.84-0.92)	<0.001	0.72	
L (G/L)*	0.10 (0.05-0.24)	<0.001	0.46	
TB (µmol/L)*	1.125 (1.047-1.209)	0.001		
TNI (ng/L)*	1.113 (1.048-1.181)	<0.001	**3.87*^a^***	**0.74**
CysC (mg/L)*	3.079 (1.441-6.579)	0.004	0.33	
A/G*	0.031 (0.008-0.114)	<0.001	0.43	
ALB (g/L)*	0.800 (0.738-0.867)	<0.001	0.32	
Ca (mmol/L)*	0.001 (0.000-0.012)	<0.001	**0.82*^a^***	**-0.38**
CK (U/L)*	1.007 (1.003-1.012)	0.002	0.45	
LDH (U/L)*	1.012 (1.007-1.017)	<0.001		
ESR (mm/h)*	1.024 (1.007-1.041)	0.005		
PCT (µg/L)*	1.2e4 (22-7.4e6)	0.004	**1.19*^a^***	**0.96**
CRP (mg/L)*	1.061 (1.038-1.085)	<0.001	**1.17*^a^***	**1.13**
IL-6 (pg/ml)*	1.115 (1.074-1.157)	<0.001	**11.97*^a^***	**2.13**
IL-10 (pg/ml)*	1.474 (1.166-1862)	0.001		

**Note.** *Per 1 for unit increase. The regression coefficient indicated the weights of the selected features in the multivariate logistic regression with L1 regularization and feature standardization. The parameter *C* had a value of 3.999 in this regression with L1 regularization. *a:* The features that were chosen for a prognostic prediction of severity for patients with moderate COVID-19. The feature weight indicated the weight for each feature used in the probability calculation formula for the prediction of severity in the prognostic model of a linear kernel SVM classifier.

## Abbreviations

COVID-19: Coronavirus Disease 2019
ICU: intensive care unit
ARDS: acute respiratory distress syndrome
WHO: World Health Organization
RT-PCR: quantitative real-time reverse transcription polymerase chain reaction
COPD: chronic obstructive pulmonary disease
HIV: human immunodeficiency virus
SVM: support vector machine
IQR: interquartile range
SD: standard deviation
WBC: white blood cell
N: neutrophil
AST: aspartate aminotransferase
LDH: lactate dehydrogenase
TNI: high-sensitivity cardiac troponin I
CK: creatine kinase
CK-MB: creatine kinase-MB
CysC: cystatin C
ESR: erythrocyte sedimentation rate
CRP: high-sensitivity C-reactive protein
PCT: procalcitonin
IL: interleukin
L: lymphocyte
A/G: albumin to globulin ratio
ALB: albumin concentration
Ca: calcium
ROC: receiver operating characteristic curve
Hb: hemoglobin
PLT: platelet count
TB: total bilirubin
ALT: alanine aminotransferase
GLB: globulin
ALP: alkaline phosphatase
BUN: blood urea nitrogen
CREA: creatinine
TG: triglyceride
Na: sodium
K: Potassium
TNF: Tumor necrosis factor-α
NK: natural killer cell
Ig: Immune globulin
ROC: Receiver operating characteristic
AUC: area under curve
